# The Nursing in Fever Hospitals

**Published:** 1889-08-17

**Authors:** 


### The Nursing in Fever Hospitals.

*(From a Correspondent.)*

As some of your readers must be aware, there is a belief amongst nurses that nursing in fever hospitals is below the average, and that the care given to infectious cases who have to seek relief in a hospital is not so thorough as that given in large general hospitals. The outside public know very little about what goes on in the wards of a fever hospital; for their own good they are refused admission to these dangerous precincts, and "knowing nothing, they have all to dread." Doubtless the absence of visitors does make the patients more dull, but the real reason of the complaints that have circulated in hospital circles undoubtedly arose from the strain on the resources of the fever hospitals caused by the scarlet-fever epidemic of 18 months ago. During that time many nurses and doctors found themselves in the position of patients; and it must be confessed that very bad patients they were! The fact is that to procure fever nurses at all at that time was a difficulty, and many of those who undertook the work were both young and untrained. And those young creatures had to nurse old charge nurses, or sisters from Bart's. or St. Mary's!

Several of the prevalent complaints, however, dealt, not with the nurses' doings, but with the general ordering of the fever hospitals. For instance, scarlet-fever patients, with temperatures ranging in the hundreds, did not appreciate getting up to have their beds made; but, say the physicians, this is no ground for complaint; there is no reason why a scarlet-fever patient with a temperature of 104 degrees should not get up while her bed is made. Therefore, those nurses who grumbled on this score merely betrayed their ignorance of medical matters. Other complaints of draught and cold are treated in the same summary manner; the "cold theory" is the modern cure for scarlet-fever, and a patient must not object to the treatment pursued.

The trials of nurses in fever hospitals are many and great; they are to a certain extent cut off from their friends, and they ought, therefore, to be better paid, better fed, and supplied with more rest and relaxation. The consideration they need is seldom theirs, and it is scarcely to be wondered at if some of them do not belong to the highest class of their profession. In some of the infectious hospitals in country districts the nurses have to cook their own food, and lead such isolated lives that the only wonder is any women are found to accept such posts. Even in the large and well-managed fever hospitals the nurses are not instructed with all the care they might be, and lectures and classes are few and far between. To grumble at these nurses, then, is an ungrateful act of their fellow-workers, and shows a decided want of sympathy and kindly feeling.

To sum up, the complaints about the fever hospitals are twofold. Those about the carelessness of nurses arose from the necessity of taking young untrained women into service during the late epidemic, and do not apply to present days. Those about general arrangements arose from ignorance of the fact that certain physicians see no reason why scarlet-fever patients with a temperature of over 100deg. should not get out of bed and sit in a draught.

[We publish the foregoing notes because we have heard much of these complaints, and having taken some pains to investigate the matter, we find them to be largely if not entirely unfounded. We welcome the opportunity thus afforded of dealing with this question generally, and hope to hear no more of such complaints in the future.—Ed. *T. H.*]

